# Curcumin mitigates cerebral vasospasm and early brain injury following subarachnoid hemorrhage via inhibiting cerebral inflammation

**DOI:** 10.1002/brb3.790

**Published:** 2017-08-09

**Authors:** Jun Cai, Dandan Xu, Xiaoxin Bai, Ruihuan Pan, Bei Wang, Shuangxi Sun, Ruicong Chen, Jingbo Sun, Yan Huang

**Affiliations:** ^1^ Diagnosis and Treatment Center of Encephalopathy Guangdong Provincial Hospital of Chinese Medicine Guangzhou China; ^2^ Department of Neurosurgery Hospital of Guangzhou University Mega Center Guangdong Provincial Hospital of Chinese Medicine Guangzhou China; ^3^ Department of Rehabilitation Hospital of Guangzhou Higher Education Mega Center Guangdong Provincial Hospital of Chinese Medicine Guangzhou China

**Keywords:** cerebral vasospasm, curcumin, early brain injury, inflammation, NF‐κB, subarachnoid hemorrhage

## Abstract

**Background and Purpose:**

Subarachnoid hemorrhage (SAH)‐induced cerebral vasospasm and early brain injury is a fatal clinical syndrome. Cerebral vasospasm and early brain injury are associated with inflammatory response and oxidative stress. Whether curcumin, which plays important roles to regulate inflammatory cytokines and inhibit oxidative stress, inhibits SAH‐induced inflammation and oxidative stress are largely unknown.

**Methods:**

Adult male rats underwent autologous blood injection into prechiasmatic cistern to induce SAH. Curcumin (150 mg/kg) was administered at 0.5, 24 and 48 hr post‐SAH. Mortality calculation and neurological outcomes as well as morphological vasospasm of anterior cerebral artery were studied. Superoxide dismutase, lipid peroxidation, and inflammatory cytokines (MCP‐1 and TNF‐α) expression in prefrontal region were quantified. Furthermore, p65 and phosphor‐p65 were quantitatively analyzed.

**Results:**

Curcumin remarkedly reduced mortality and ameliorated neurological deficits after SAH induction (*p* < .05); morphological results showed that cerebral vasospasm in curcumin‐treated group was mitigated (*p* < .05). SAH‐induced MCP‐1 and TNF‐α overexpression were inhibited in curcumin‐treated group (*p* < .05). Importantly, phosphor‐p65 was significantly inhibited after curcumin treatment (*p* < .05).

**Conclusions:**

Curcumin can inhibit SAH‐induced inflammatory response via restricting NF‐κB activation to alleviate cerebral vasospasm and early brain injury.

## 1 INTRODUCTION

Subarachnoid hemorrhage (SAH), which is mostly resulted from rupture of cerebral aneurysm, is a high mortality and morbidity devastating condition, accounting for 5% of all strokes and affecting 2 in 100,000 Chinese people annually (over 30,000 in total) (Bederson et al., 2009; Ingall, Asplund, Mahonen, & Bonita, 2000). Early brain injury (EBI) and cerebral vasospasm (CV) are considered the leading causes of death and disability in patients suffering SAH (Macdonald, Pluta, & Zhang, 2007; Sehba, Pluta, & Zhang, 2011; Suarez, Tarr, & Selman, 2006; Wang et al., 2012). EBI is the product of pathological mechanism triggered by oxidative stress, inflammation, cell death and so on, the mechanism of which needs to be elucidated (Hasegawa, Suzuki, Sozen, Altay, & Zhang, 2011; Sehba et al., 2011). Increasing evidence presented that inflammatory response and oxidative stress were involved in the mechanism of CV following SAH (Laban et al., 2015; Zhao, Wen, Dong, & Lu, 2016). These two devastating injured mechanisms were assessed in this study.

Curcumin (diferuloylmethane) is an active component of turmeric derived from the root of the Curcuma longa Linn. Due to its multiple properties of anti‐inflammatory, antioxidant, anti‐apoptosis, and anticancer, curcumin was used in several preclinical and clinical trials, such as in cancer, atherosclerosis, aging, neurodegenerative disease, hepatic disorders, obesity, diabetes, AIDS, psoriasis, and autoimmune diseases (Shishodia, 2013). In addition, it displayed diverse and significant neuroprotective effects in experimental researches, such as ischemic stroke, traumatic brain injury, and intracranial hemorrhage (Jiang et al., 2007; Sun et al., 2011; Wang, Gu, Qin, Zhong, & Meng, 2013; Wu, Ying, & Gomez‐Pinilla, 2006). The therapeutic effects of curcumin have been confirmed after SAH. Curcumin was reported to be capable of decreasing mortality and attenuating oxidative stress and cerebral vasospasm (basilar artery) following SAH induction (Kuo et al., 2011; Wakade, King, Laird, Alleyne, & Dhandapani, 2009; Yuan et al., 2017; Zhang, Kong, Wang, Xu, & Zhu, 2016). However, more information detailing neuroprotective abilities of curcumin following SAH need to be delineated. We designed present study to observe the protective effects of curcumin in SAH rats. We also evaluated the changes of inflammatory factors, oxidative stress, and transcriptional factors during the course of SAH induction; and assessed the connections of these neurotoxic factors and curcumin administration.

## 2 METHODS

### 2.1 Animal groups and study design

Animal procedures were carried out according to a protocol approved by the Institutional Animal Care and Use Committee (IACUC) at Guangdong Provincial Hospital of Chinese Medicine, Guangzhou, China. One hundred and eighty‐four Sprague–Dawley rats (Medical Laboratory Animal Center of Guangdong, Guangzhou, China) weighing 300–350 g (12–14 weeks) were used in this study. The rats, which were housed in the animal room at 22–24°C with 12‐hour light/dark circle and free access to food and water, were randomized into sham group (*n* = 36), SAH group (SAH, *n* = 55), vehicle group (VEH, *n* = 51), and curcumin group (CUR, *n* = 42).

Rats from SAH, VEH, and CUR groups were underwent SAH induction; while animals from sham group were subjected to sham operation. Mortality rates were calculated for all groups 48 hr after SAH induction.

The sham operation consisted of calvarium incising, skull burr hole drilling, and autologous blood collecting; instead of injecting autologous blood into prechiasmatic cistern. Following blood injection, rats of curcumin group or vehicle group were intraperitoneally injected with 150 mg/kg curcumin (dissolved into 10% dimethyl sulfoxide solution) or equal volume 10% dimethyl sulfoxide solution. Mortality calculation and neurological deficit assessment were performed 48 hr after SAH induction. Following neurobehavioral evaluation, all rats were killed for histological measurement, oxidative stress evaluation, and molecular analysis.

**Figure 1:**
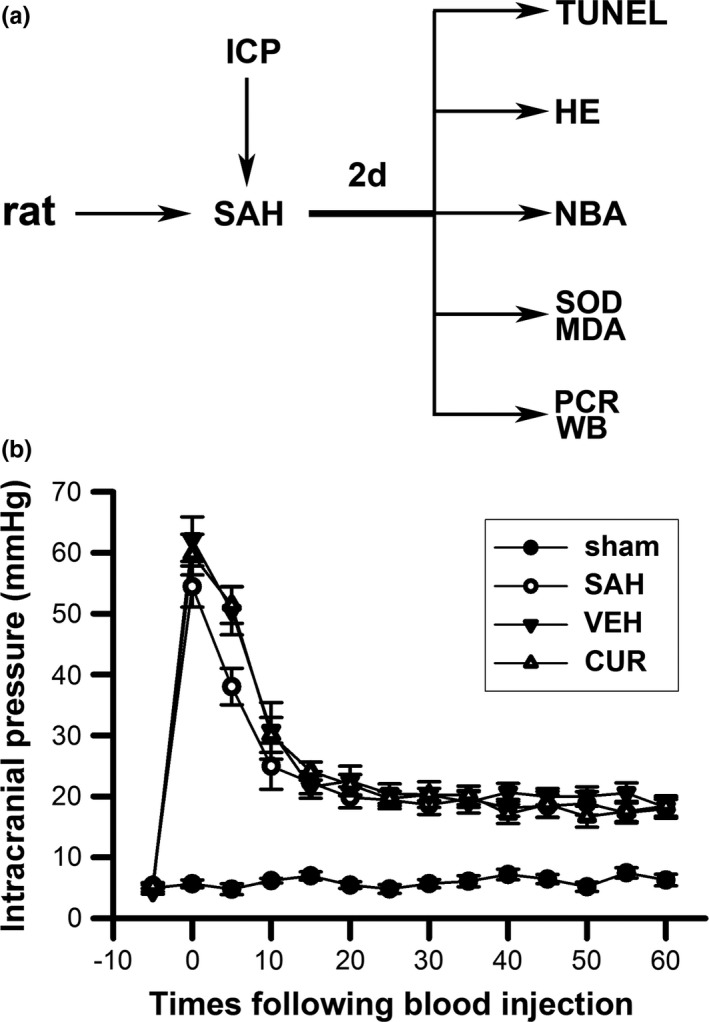
Study design of present study (a) and the changes of intracranial pressure during subarachnoid hemorrhage (SAH) induction (b). (a) Intracranial pressure (ICP) of animals were monitored during SAH induction. Rats from all groups were subjected to NBA (neurobehavioral assessment), histological measurement (HE), superoxide dismutase, malondialdehyde measurement, TUNEL staining, PCR, and western blot (WB) analyses 2 days after SAH induction. (b) Line graphs display ICP changes of four animal groups during nonheparin autologous blood injecting into prechiasmatic cistern. ICP of sham group maintain at a stable level; ICP of SAH, vehicle, and curcumin groups remarkedly increased immediately after blood injection, and, slowly decreased with time. Data are means ± *SEM*; n = 5. SAH, subarachnoid hemorrhage

The basic sample sizes of rats were 36 each group for neurobehavioral assessment, histological measurement, oxidative stress evaluation, TUNEL assay, quantitative real‐time PCR analyses, and western‐blot analyses. The rats from all groups were randomly distributed for neurological deficits assessment (*n* = 8), histological measurement (*n* = 6), determination of superoxide dismutase (SOD) activity, malondialdehyde (MDA) content (*n* = 6), quantitative real‐time PCR (*n* = 6), TUNEL staining (*n* = 5) and western‐blotting (*n* = 6). To make up for the loss following SAH induction, we augmented the sample sizes of some groups. In addition, all animals were included for statistical analyses in this study. The study design is displayed in Figure 1a.

### 2.2 Induction of SAH

The rats from all groups were anesthetized via chloral hydrate (35 mg/kg) ip. After anesthesia, a heating pad (RWD Life Science Co.; Shenzhen, China) was used to maintain animal temperature at 37.0 ± 0.5°C. Nonheparin blood was extracted from left femoral artery. Rats were fixed in a stereotactic frame (RWD Life Science Co.; Shenzhen, China) and placed in a prone position. The modified protocol of induction of SAH has been previously reported (Cai et al., 2012, 2013); and a brief description is as follows: A burr hole at 0.5 mm right away from midline and 7.5 mm anterior to bregma was drilled. Autologous blood was collected into a 1 ml syringe with a 27‐gauge needle, which was controlled by a micro‐injecting pump (World Precision Instruments Inc., Sarasota, FL, USA). The 27‐gauge needle was inserted into the hole and tilted 30° in the sagittal plane; if the needle was into prechiasmatic cistern, transparent cerebrospinal fluid (CSF) was back‐flowed into the syringe. Then, 200 μl nonheparin, autologous blood was injected into prechiasmatic cistern mechanically. A multipurpose physiological apparatus (PowerLab, AD‐instrument Co., Australia) was used to continuously monitor intracranial pressure (ICP) changes during SAH induction, as reported previously (Barth, Onesti, Krauss, & Solomon, 1992; Cai et al., 2012, 2013).

### 2.3 Mortality calculation and neurological deficit assessment

Mortality calculation of all groups was performed 48 hr after SAH induction. After mortality calculation, the rats of all groups were subjected to neurobehavioral evaluation by a blind observer via an 18‐point system (Garcia, Wagner, Liu, & Hu, 1995).

### 2.4 Histological measurement

The caliber and wall thickness of anterior cerebral artery (ACA) were measured in hematoxylin and eosin (HE) staining slices; HE staining was performed through a commercial kit (Beyotime Biotechnology; Jiangsu, China). Following euthanasia, the brains were fixed in 4% paraformaldehyde for 24 hr. Brain samples vertical to ACA (Data S1) were collected via an operating microscope (Carl Zeiss AG; Heidenheim, Germany) as reported previously (Cai et al., 2012, 2013). The 4‐μm thick slices, which were stained with HE, were viewed under a light microscope (Leica Co., Wetzlar, Germany). Caliber and wall thickness of ACA were measured at the bifurcation of ACA and Olfactory artery by an observer blindly using the Image J software (NIH Program; Bethesda, MD, USA). The caliber and wall thickness of ACA in all groups were averaged from several brain samples (*n* = 6).

### 2.5 Determination of SOD activity and MDA content

The SOD activity in the area of frontal lobe, which was adjacent to blood injection site, was measured according to xanthine oxidase method via a standard assay kit (Nanjing Jiancheng Bioengineering Institute, #A001‐3, PubMed: 24505260; Nanjing, China). The xanthine‐xanthine oxidase system was used to produce superoxide ions, which reacted with 2‐(4‐iodophenyl)‐3‐(4‐nitrophenol‐5‐phenlyltetrazolium chloride). The rate of the reduction with O2 was linearly related to the xanthine oxidase (XO) activity, and is inhibited by SOD; therefore, the value of SOD activity in frontal lobe could be defined via spectrophotometric measurement.

Lipid peroxidation was evaluated by measuring MDA concentrations according to the thiobarbituric acid (TBA) method through a standard assay kit (Nanjing Jiancheng Bioengineering Institute, #A003‐1, PubMed: 24058471). The principle of this assay was based on the reaction of TBA and MDA, which could be determined via spectrophotometric measurement.

These abovementioned procedures were performed according to the manufacturer's instructions. The values of SOD and MDA in all groups were averaged from several brain samples (n = 6).

### 2.6 Quantitative real‐time PCR

We evaluated inflammatory response in SAH rats via measuring the mRNA of Monocyte Chemoattractant Protein‐1 (MCP‐1) and tumor necrosis factor‐α (TNF‐α). The levels of MCP‐1 and TNF‐α mRNA were determined by TaqMan real‐time PCR (n = 6). Total RNA was isolated from frontal lobe with Trizol reagents (Invitrogen, #15596018, PubMed:12411577; Carlsbad, CA, USA) according to the manufacturer's instructions. A PrimeScript RT reagent kit (TaKaRa Bio; Shiga, Japan) was used for synthesizing cDNA templates from the total RNA. The primers were synthesized by Yingjun Biotechnology (Shanghai, China) and are shown in Table 1. Quantitative real‐time PCR was proceeded through a model 7700 sequence detector (PE Applied Biosystems; Chiba, Japan) via a TaqMan PCR reagent kit (Invitrogen, #4392938, PubMed:22885101). The experiments were repeated twice.

**Table 1:** The primers of MCP‐1 and TNF‐α used in this study

Target genes	Sense primer (5′–3′)	Antisense primer (5′–3′)	Annealing temperature (°C)	Number of cycles	Size (bp)
MCP‐1	GCTTCTGGGCCTGTTGTTCAC	CACAGATCTCTCTCTTGAGCTTG	60	40	1228
TNF‐α	GTGATCGGTCCCAACAAGGAG	GTCTTTGAGATCCATGCCATTGG	62	40	1142
β‐Actin	CACCCGCGAGTACAACCTTC	GACCCATACCCACCATCACAC	61	40	209

### 2.7 Terminal deoxynucleotidyl transferase‐mediated dUTP nick end labeling staining and apoptotic cells calculation

TUNEL technique was used to detect cell apoptosis in frontal lobe via an *in situ* cell death detection kit (Roche Diagnostics GmbH, Roche Applied Science, #12156792910 PubMed: 24831012; Penzberg, Germany) according to the protocol of the kit. An immunofluorescence analysis of neurons with antibody against the neuronal marker protein‐NeuN (Millipore, #MAB377, PubMed: 26373451; Billerica, MA), TUNEL staining, and DAPI (Molecular Probes, #D1306, PubMed: 11500852; Eugene, OR) staining were performed in specific area of frontal lobes (Figure 4b) to calculate TUNEL‐positive cells. In each section of rat brain, three nonoverlapping visual fields were chosen randomly within the regions of interest. A minimum of 300 cells were counted, and those cells with NeuN, TUNEL‐positive, and intense chromatin clumping (DAPI staining) were counted as neuronal apoptotic cells. The positive cells were distinguished, counted, and analyzed under a light microscope by an observer blinded to the study. The results of apoptotic cells calculation were averaged from several rats in each group (*n* = 5).

### 2.8 Western blotting

The frozen brain samples were homogenized with RIPA buffer (Cell Signaling Technologies, #9806, pubmed: 20581862; Beverly, MA, USA) containing protease and phosphatase inhibitor cocktail (Roche Diagnostics GmbH, Roche Applied Science, #05892791001 & #04906837001, PubMed: 22268099 & 22453918; Penzberg, Germany); and, lysed with a buffer containing 20 mmol/L Tris (pH 7.6), 0.2% SDS, 1% TritonX‐100, 1% deoxycholate, 1 mmol/L phenylmethylsulfonyl fluoride, and 0.11 IU/ml aprotinin. All the ingredients were purchased from Sigma‐Aldrich. The total protein was extracted from the brain samples and subjected to an 8% SDS‐PAGE (n = 6). The following antibodies were used: polyclonal antibodies against MCP‐1 (1:1,000 dilution; Abcam, #ab25124,PubMed: 21750230; Cambridge, MA, USA), TNF‐α (1:1,000 dilution; Abcam, #ab6671,PubMed: 26408546), P65 (1:1,000 dilution; Cell Signaling Technology, #6956S, Clone No. L8F6, PubMed: 27716383; Beverly, MA, USA), and phosphorylated P65 (1:1,000 dilution; Cell Signaling Technology, #3033S, Clone No. 93H1, PubMed: 28165507). We chose β‐actin (1:6,000 dilution; Sigma‐Aldrich, #A5441, Clone No. AC‐15, PubMed: 28276506; St Louis, MO, USA) as the secondary antibody. The densitometry analyses of the western blots were performed with Glyko Bandscan software (Glyko; Novato, CA, USA). The experiments were conducted at least three times.

### 2.9 Statistical analyses

The data are shown as the means ± the standard error of the means (SEMs). A one‐way analyses of variance (ANOVA) followed by a Student, Newman–Keuls or Dunnett's *post hoc* test were utilized for the comparisons between more than two groups. Besides, mortality rate was compared by chi‐square test. SPSS 18.0 (SPSS, Chicago, IL, USA) was used for the statistical analyses, and the statistical significance was set at *p *<* *.05.

## 3 RESULTS

### 3.1 ICP monitor confirmed the uniformity of SAH models in all groups

The ICP changes of all animals were recorded during SAH induction (The ICP values of 5 min before SAH induction and 5, 10, 15, 20, 25, 30, 35, 40, 45, 50, 55, 60 min after SAH induction were recorded). We displayed the ICP changes (n = 5) during blood injection in Figure 1b. According to the ICP changes, the uniformity of SAH models from all SAH induction groups was verified.

### 3.2 Curcumin decreased mortality and ameliorated neurological deficits following SAH induction

**Figure 2:**
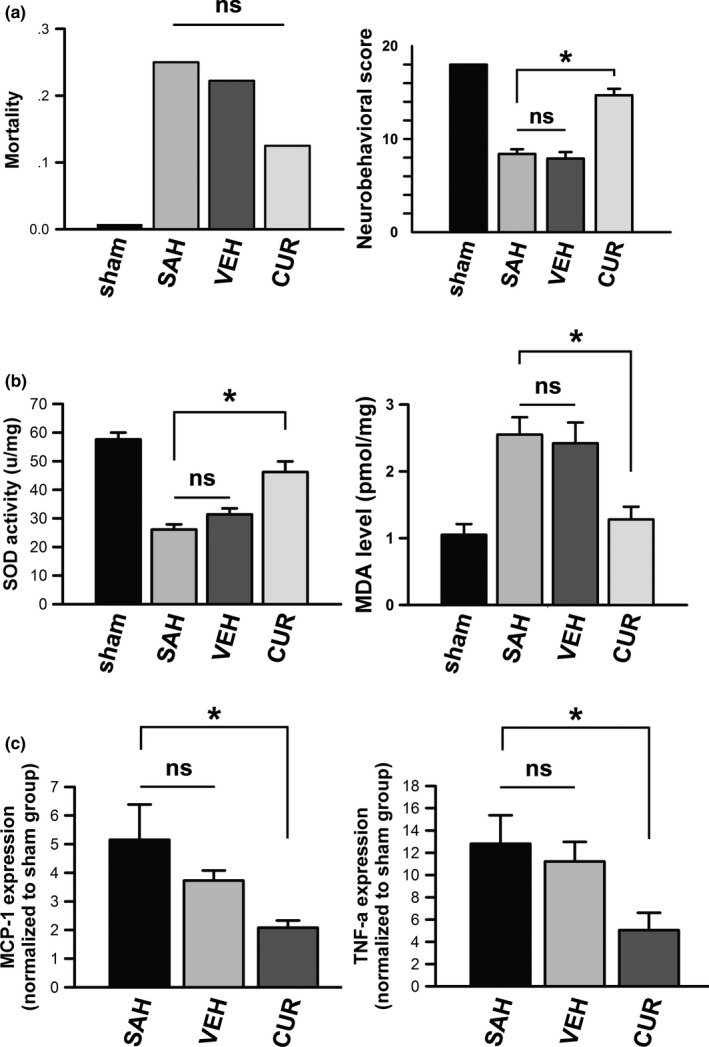
The differences in mortality, neurological deficits assessment, oxidative stress evaluation, and proinflammatory cytokines of all groups. (a) Mortality and neurobehavioral scores of all groups are displayed as bar graphs. Data are means ± *SEM*; n = 8; *p <* *.05; ns, not significant. (b) Superoxide dismutase activities and malondialdehyde levels of all groups are shown in bar graphs. Data are means ± *SEM*; n = 6; *p <* *.05; ns, not significant. (c) mRNA activities of proinflammatory cytokines of MCP‐1 and TNF‐α in all groups are shown in bar graphs. Data are means ± *SEM*; n = 6; *p <* *.05; ns, not significant

We calculated the mortality of all groups 48 hr after blood injection; the mortality of sham, SAH, VEH, and CUR groups were 0 (0/36), 25% (14/55), 22% (11/51), and 13% (5/42), respectively, which is displayed in Figure 2a. Mortality in CUR group was lower than other SAH induction groups (SAH and VEH groups). However, there was no significant difference between SAH and CUR groups with chi‐square test (*p *>* *.05).

Neurological deficits assessment was performed following mortality calculation. According to an 18‐point neurobehavioral score system, The values of neurobehavioral scores in sham, SAH, VEH, and CUR groups were 18, 8.4 ± 0.5, 7.9 ± 0.7 and 14.7 ± 0.7, respectively. The rats of sham group did not suffer any neurological deficits. Rats from SAH, VEH, and CUR groups presented neurological dysfunction compared to sham group (*p *<* *.05). However, the value of neurobehavioral evaluation (Figure 2a) disclosed CUR group suffered minor neurological deficits compared to SAH and VEH groups (*p *<* *.05).

### 3.3 Curcumin ameliorated cerebral vasospasm after SAH induction

**Figure 3:**
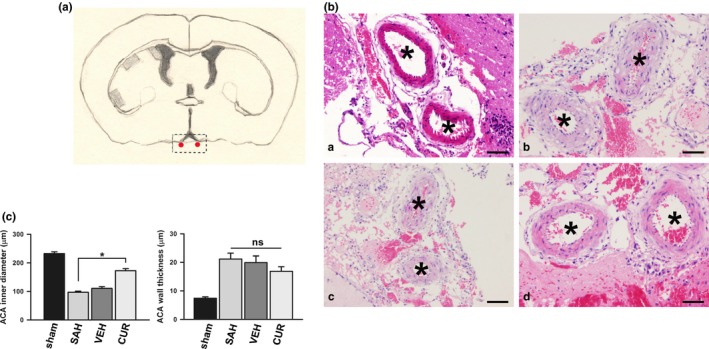
Anterior cerebral artery (ACA) photomicrographs. (A) A sketch shows coronal section of rat brain, and, points out the portions in which ACA samples are collected using dotted square. (B & C) ACA photomicrographs with HE staining display ACA cross sections (including vessel caliber and vessel wall thickness) of sham (a), subarachnoid hemorrhage (b), vehicle (c), and curcumin (d) groups. Data are means ± *SEM*; n = 8; *p <* *.05; ns, not significant. Scale bar = 20 μm

Inner diameter and vessel wall thickness of ACA were measured on HE stained slices to evaluate cerebral vasospasm. The average inner diameters of sham, SAH, VEH and CUR groups were 232.3 ± 6.44, 96.9 ± 4.11, 110.5 ± 6.24, and 172.6 ± 7.23 μm, respectively. The average vessel wall thickness of these four groups was 7.4 ± 0.49, 21.2 ± 2.05, 19.9 ± 2.32, and 16.9 ± 1.62 μm, respectively. Inner diameter of ACA in CUR group was remarkedly larger than that in SAH group (*p *<* *.05). Vessel wall thickness in CUR group was smaller than that in SAH group (*p *>* *.05). Cross section of normal ACA was displayed in Figure 3Ba; cross section of typical vasospastic ACA was showed in Figure 3Bb; ACA cross section of curcumin‐treated was exhibited in Figure 3Bd.

### 3.4 Curcumin alleviated SAH‐induced oxidative stress

The activity of SOD was significantly activated into frontal lobes after SAH induction, as quantified by xanthine oxidase method via a standard assay kit. According to Figure 2b, SOD activity of SAH group was pronouncedly increased compared to the control (*p *<* *.05); SOD activity of curcumin‐treated rats was remarkedly limited compared to SAH‐induced rats (*p *<* *.05).

MDA is a highly reactive compound that results from lipid peroxidation of polyunsaturated fatty acids within normal in living bodies. MDA level was restrained by SAH induction. According to Figure 2b, MDA level of SAH group was prominently decreased compared to sham group (*p *<* *.05); whereas, to some extent, curcumin treatment could restored MDA level compared to SAH‐induced rats (*p *<* *.05).

### 3.5 Curcumin limited tissue inflammation induced by SAH

MCP‐1 and TNF‐α were used to reflect inflammatory response in the area of frontal lobe in this study. The mRNA levels of MCP‐1 and TNF‐α were remarkedly increased by SAH induction according to Figure 2c, compared to sham group (*p *<* *.05). However, the increasing mRNA expressions of MCP‐1 and TNF‐α could be limited by curcumin treatment (*p *<* *.05).

The protein quantities of MCP‐1 and TNF‐α were also analyzed via western blot technique. According to Figure 5a, b, the protein expressions of MCP‐1 and TNF‐α were significantly increased by SAH induction compared to sham group (*p *<* *.05). MCP‐1 and TNF‐α were quantitatively decreased in curcumin‐treated group compared to SAH group (*p *<* *.05).

### 3.6 Curcumin reduced cell apoptosis after SAH induction

**Figure 4:**
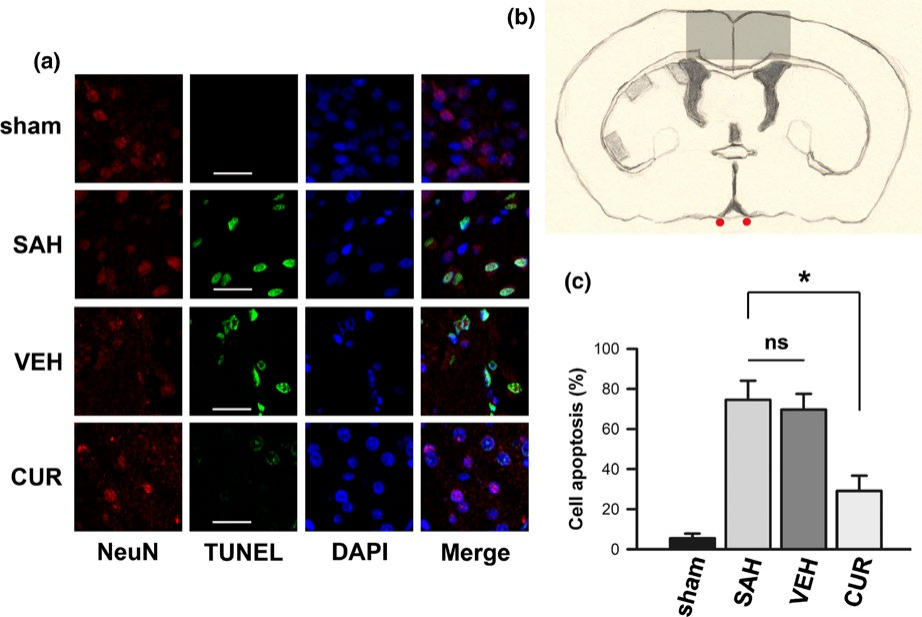
Terminal deoxynucleotidyl transferase‐mediated dUTP nick end labeling (TUNEL) staining in frontal lobe. (a) Fluorescent photomicrographs exhibit typical TUNEL staining of frontal lobe in all groups. (b) A sketch is used to delineate the portions (shade), which were adopted to observe and calculate apoptotic neurons. (c) Bar graphs show subarachnoid hemorrhage‐induced cell apoptosis is restrained by curcumin treatment. Data are means ± *SEM*; n = 5; *p <* *.05; ns, not significant

After autologous blood injecting into prechiasmatic cistern, apoptotic cells remarkedly increased in frontal lobe according to Figure 4a,c (*p *<* *.05). In normal status, the apoptotic rate was about 5% (sham group); however, the apoptotic rate pronouncedly increased to 74% following SAH induction (SAH group). Otherwise, curcumin treatment could restored the apoptotic rate to 29%, which was significantly less than SAH group (*p *<* *.05).

### 3.7 Curcumin restrained SAH‐activated NF‐κB

**Figure 5:**
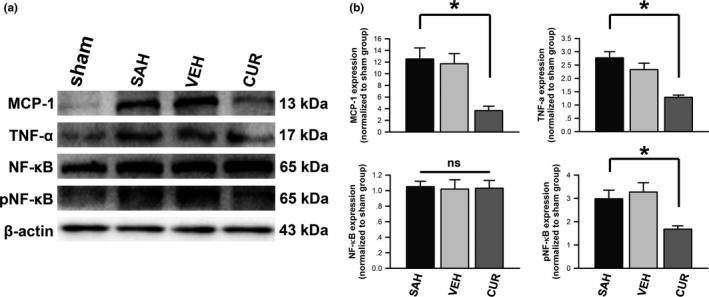
Expressions of proinflammatory cytokines and P65 in frontal lobe. (a) Representative autoradiogram of MCP‐1, TNF‐α, total P65, and phosphorylated P65 expressions measured by western blot are displayed. (b) The proinflammatory cytokines and phosphorylated P65 are displayed in bar graphs. Data in subarachnoid hemorrhage, vehicle, and curcumin groups are normalized to sham group. Data are means ± *SEM*; n = 6; *p <* *.05; ns, not significant

NF‐κB as a transcription factor that participates in various biological processes, including inflammation. P65 was chosen to be assessed in this study. The protein quantity of P65 was analyzed via western blot technique. Total P65 in frontal lobe of rats from all groups were similar (*p *>* *.05). Compared to the control, phosphorylated P65 was remarkedly increased after SAH induction (Figure 5); however, increased P65 expression could be inhibited in curcumin‐treated group (Figure 5; *p *<* *.05).

## 4 DISCUSSION

Including prechiasmatic cistern injection model, common mortality of experimental SAH rats were various from 9% to 100% (Gules, Satoh, Clower, Nanda, & Zhang, 2002; Lee, Huang, Keep, & Sagher, 2008; Lee, Sagher, Keep, Hua, & Xi, 2009; Park et al., 2008; Prunell, Mathiesen, & Svendgaard, 2002). In present study, the mortality of SAH and VEH group were 25% and 22%, respectively, which were similar to other studies using this injection model (Boettinger et al., 2017; Dou et al., 2017). However, about 87% of the rats treated with curcumin survived from SAH induction (The mortality of curcumin‐treated rats were 13%). In addition, neurological deficits assessment disclosed that the rats treated with curcumin suffered less neurological deficits, compared to the SAH rats. We conclude that following SAH induction, mortality and neurological assessment were two parallel parameters, less mortality less neurological loss. And, we identified that curcumin treatment after SAH induction could effectively ameliorate neurological loss as well as decrease mortality.

TNF‐α and MCP‐1 are both proinflammatory cytokines that are associated with oxidative stress, cell death, and recruitment of inflammatory mediators (Niwa et al., 2016; Przybycien‐Szymanska & Ashley, 2015). A number of inflammatory mediators were confirmed to be up‐regulated after SAH include TNF‐α and MCP‐1 (Dumont et al., 2003; Rahmanian et al., 2015; Wu et al., 2016). The mRNA and protein expressions of TNF‐α and MCP‐1 were detected to be remarkedly increased 2 days after SAH induction in this study (Figures 2 and 5). A prospective clinical study reported that the elevated TNF‐α expression in serum on 2–3 days after aneurismal SAH was correlated with poor clinical outcomes (Chou et al., 2012). We found that curcumin treatment inhibited mRNA and protein expressions of TNF‐α and MCP‐1.

It was reported that the pathophysiologic consequences of an aneurysmal SAH led not only to vasospasm but also to a global ischemic injury to the brain; this kind of brain injury is called EBI (Cahill & Zhang, 2009). The pathophysiologic changes of EBI are comprised of cell death (necrosis, apoptosis and autophagy), oxidative stress, and inflammatory response (Sehba, Hou, Pluta, & Zhang, 2012). In this study, SAH‐induced neuron apoptosis (Figure 4) and oxidative stress (Figure 2b) could be attenuated by curcumin treatment. So essentially, curcumin is capable of ameliorating SAH‐induced EBI.

**Figure 6:**
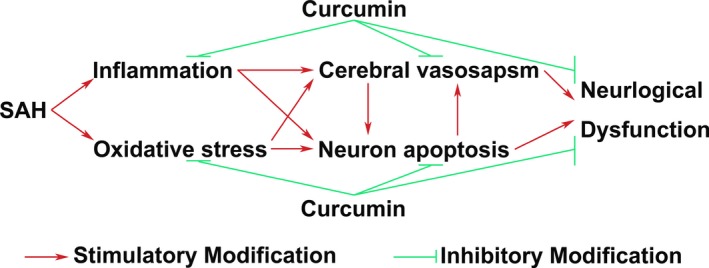
A sketch summarizing present study. In this study, we have found that curcumin can markedly mitigate cerebral vasospasm and early brain injury. And, neurological deficits following subarachnoid hemorrhage (SAH) have been ameliorated after curcumin treatment. In the meanwhile, proinflammatory factors, inflammatory transcription factor (P65), and oxidative stress have been restricted by curcumin treatment. We postulated that curcumin was capable of inhibiting SAH‐induced inflammatory response and oxidative stress to alleviate cerebral vasospasm and early brain injury

P65 is one of the NF‐κB family of transcription factors, which participates in various biological processes, including immune response, inflammation, development, cell growth, and survival (Hayden & Ghosh, 2008; Vallabhapurapu & Karin, 2009). As one of the most abundant and well‐characterized NF‐κB dimer, P65 (RelA) translocate to nuclear and drive transcriptional response (Hayden & Ghosh, 2008; Karin & Ben‐Neriah, 2000; Vallabhapurapu & Karin, 2009). Phosphorylated form of P65/RelA is specifically required to activate transcriptional responses, including inflammatory response (Jamaluddin, Wang, Boldogh, Tian, & Brasier, 2007; Nicodeme et al., 2010; Nowak et al., 2008; Vermeulen, De Wilde, Van Damme, Vanden Berghe, & Haegeman, 2003; Zhong, SuYang, Erdjument‐Bromage, Tempst, & Ghosh, 1997). In this study, phosphorylated P65 was detected to be significantly up‐regulated following SAH induction. The inhibitory modification of the inflammatory mediators (TNF‐α and MCP‐1) was consistent with restraint of P65 activation after curcumin treatment following SAH induction. In addition, SAH‐induced pathophysiologic changes of EBI were ameliorated by curcumin treatment. We postulated that curcumin might inhibit NF‐κB canonical pathway to attenuate SAH‐induced CV and EBI (Figure 6).

To alleviate SAH‐induced CV, a number of medications have been tested in clinic and laboratory. Nimodipine, a dihydropyridine calcium channel blocker, is the most widely used and the only effective pharmacologic intervention to prevent and cure CV in practice (Francoeur & Mayer, 2016). Nonetheless, there was no definite connection with angiographic alleviation and functional improvement (Laskowitz & Kolls, 2010). Moreover, radiographic vasospasm improvement lacks evidence to declare its neural functional amelioration in CV (Polin et al., 2000). It should be pointed out that EBI may be the primary cause of mortality in SAH patients (Cahill & Zhang, 2009). Curcumin may be a promising endeavor in near future.

In summary, curcumin might be capable of inhibiting SAH‐induced inflammatory response via restricting NF‐κB activation to alleviate cerebral vasospasm and early brain injury.

## CONFLICT OF INTEREST

The authors have no actual and potential competing interests to declare.

## AUTHORs’ CONTRIBUTIONs

All authors had full access to all the data in the study and take responsibility for the integrity of the data and the accuracy of the data analysis. Conceived and designed the experiments: JC, YH. Performed the experiments: DX, JC, RP, SS. Analyzed the data: XB, RC. Contributed reagents/materials/analysis tools: JS, YH, XB, JC. Wrote the paper: JC. Revised the manuscript: YH, JS, JC.

## AVAILABILITY OF DATA AND MATERIALS

The datasets used and/or analyzed during this study is available from the corresponding author on reasonable request.

## SIGNIFICANCE STATEMENT

According to the results of present study, we demonstrated that curcumin could inhibit subarachnoid hemorrhage (SAH)‐induced inflammatory response via restricting NF‐κB activation to alleviate cerebral vasospasm and early brain injury. We believe the findings will provide positive information to basic science and pharmaceutical development of SAH. We presume the findings of this study will be of special interest to the readers with background of neuroscience, clinical neurology, and neurovascular diseases.
