# Supplementary figures and images for: Curcumin mitigates cerebral vasospasm and early brain injury following subarachnoid hemorrhage via inhibiting cerebral inflammation
Source: Brain Behav. 2017 Aug 9;7(9):e00790. doi: 10.1002/brb3.790 (PMC5607553; doi:10.1002/brb3.790)

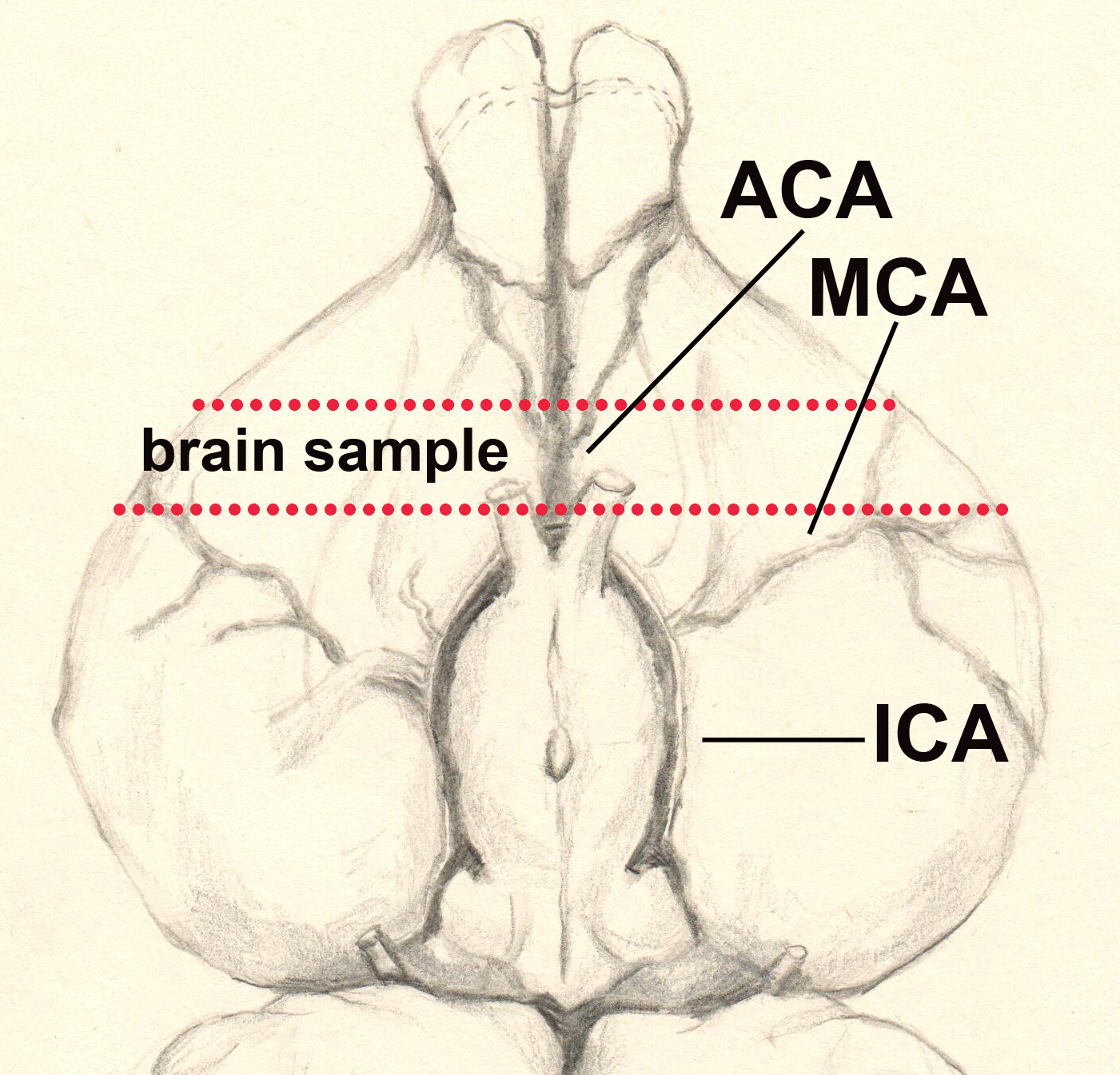

Supplement: Supplementary file 1 [file BRB3-7-e00790-s001.tif]
